# A Review of Medication Errors in Iran: Sources, Underreporting Reasons and Preventive Measures

**Published:** 2014

**Authors:** Ava Mansouri, Alireza Ahmadvand, Molouk Hadjibabaie, Mohammadreza Javadi, Seyed Hamid Khoee, Farzaneh Dastan, Kheirollah Gholami

**Affiliations:** a Research Center for Rational Use of Drugs, Tehran University of Medical Sciences, Tehran, Iran.; b Department of Epidemiology and Biostatistics, School of Public Health, Tehran University of Medical Sciences, Tehran, Iran.; c Faculty of Pharmacy, and Research Center for Rational Use of Drugs, Tehran University of Medical Sciences, Tehran, Iran.; d Clinical Pharmacy Department, School of Pharmacy, Tehran University of Medical Sciences, Tehran, Iran.

**Keywords:** Medication errors, Source of medication errors, Reporting of medication errors, Preventive measures of medication errors, Literature review

## Abstract

Medication error (ME) is the most common preventable cause of adverse drug events which negatively affects patient safety. Inadequate, low-quality studies plus wide estimation variations in ME from developing countries including Iran, decreases the reliability of ME evaluations. To clarify sources, underreporting reasons and preventive measures of MEs, we reviewed Iran current available literature. We searched Scopus, WOS, PubMed, CINAHL, EBSCOHOST and Persian databases (IranMedex, and SID) up to October 2012. Two authors independently selected and one reviewed and extracted data. Results reported by more than 30% of studies considered as the most important topics. Finally 25 articles were included. All study designs were cross-sectional (except for two interventional studies) and in hospital settings. Nursing staff and students were the most observed populations. Individual factor, with “inadequate knowledge of medication” as its most frequent reason, were the mostly reported source of MEs. Fear and reporting process were two most important reporting barriers. The sense of being reprimanded and ignoring to report respectively were their most frequent factors. Anti-infectives were the most frequent drugs involved in MEs. Preventive measures were varied and reporting of their effectiveness was inconsistent. There are still many research gaps which need to be explored by further studies. Based on our findings, further researches may be focused on design, implementation, and evaluation of a ME reporting system as groundwork, assessing systems-related factors to ME alongside individual factors and evaluating the effectiveness of preventive measures for MEs in trials.

## Introduction

Medication errors (MEs) as one of the most common types of medical problems in healthcare institutions (1, 2), are a leading cause of patient harm (3) and a worldwide concern (4, 5).

ME is defined as “a failure in the treatment process that leads to, or has the potential to lead to, harm to the patient” (6). It has been estimated that between 10% and 18% of all reported hospital harms can be attributed to MEs with varied ranges of effects, from going unnoticed to causing death (1). 

MEs may occur in any phase of medication process (4). In an update review of literature on Iranian MEs studies, prevalence of MEs in different stages were as follows: prescribing (29.8% to 47.8%), transcribing (10.0% to 51.8%), dispensing (11.3% 33.6%) and administration (14.3% to 70%) (7). Preventing MEs depends on awareness of the causes or contributing factors (3, 5); therefore, health care organizations are advised to monitor errors by establishing and promoting organization-wide reporting systems to find possible sources of ME (3). 

Evidence from ME reporting systems suggests that many MEs are not reported, for different individual and contextual reasons, and therefore go undetected (3). 

The resultant underreporting reduces the possibility of analyzing natures of MEs and developing quality improvement initiatives (8). As a developing country, Iran is no exception regarding underreporting of MEs (5) (4). Different studies have been published regarding the status and sources of MEs in Iran. But, there seems that evidence is lacking regarding the use of ME incidents in quality improvement in Iranian healthcare organizations (9). 

Moreover, many preventive strategies have been used for reducing MEs and their complications. But, the effectiveness of many of these interventions is dependent on cultural context and circumstances. So, it is better to assess the effectiveness and suitability of ME prevention strategies in Iran’s context.

MEs can occur with any medicinal product (3); but, possibly due to scarcity of resources, many studies have been focused on commonly-used products or those drugs that have been frequently involved in ME reports. So, it is expected that studies on ME from Iran may have also been focused on commonly-used drugs and their possible consequences. 

Limited number of studies on ME in developing countries makes it difficult to get a comprehensive picture on MEs (4, 5). Shortage of high-quality studies with well-designed methodologies also adds to the difficulties in providing reliable evaluation of MEs (5, 10). 

In order to give details about sources, underreporting reasons, preventive measures, and also the most common drugs associated with MEs, we planned to review current available evidence.

## Experimental

*Databases*

In order to review Persian and English language-literature on medication errors in Iran, we searched these English electronic databases to find articles related to sources, underreporting reasons, preventive measures, and also the most common drugs associated with MEs: Scopus, Web of Science, PubMed, the Cumulative Index to Nursing and Allied Health Literature (CINAHL), and EBSCOHOST. We also searched these Persian electronic databases: Iran Medex, and Scientific Information Database (SID). We additionally searched references from relevant articles to identify additional studies. The time span was up to October 2012.

*Search terms*

We used these English terms and their corresponding Persian equivalents: administration error(s), administration mistake(s), dispensing error(s), dispensing mistake(s), documentation error(s), drug mistake(s), medication error(s), medication mistake(s), nurse(s), pharmacist(s), physician(s), prescribing error(s), prescribing mistake(s), transcribing error(s), transcribing mistake(s), wrong calculation(s), wrong dose(s), wrong drug(s), wrong medication(s), and wrong route(s) of administration. Each of the words were combined using “OR” and then combined using “AND” with (Iran OR Iranian OR I.R.Iran). 

*Inclusion/exclusion criteria*

We considered all types of original studies on adults and children; *i.e*., clinical trials, cohort or case–control studies, and cross-sectional studies. We looked for studies which reported sources of MEs, reasons for not reporting MEs, preventive measures of MEs and most common drugs involved in MEs. Letters, case reports, conference papers, organizational reports, opinions or editorial papers were excluded. We also excluded articles focused on medical - not medication - errors and nursing practice errors. Moreover, we excluded articles on preventive measures which were solely focused on usability and acceptability of the measures themselves, not on the outcome of reducing MEs.

*Selection and information extraction*

Two authors independently selected and one of them reviewed the articles by following these stages: Inclusion and exclusion criteria were assessed both in reading the titles and abstracts of the search results. The data extraction tables comprised these sections which were examined in each article: sources, underreporting of, preventive measures for and drugs involved in MEs; unit of observation studied; sample size; study design and/or measurement tool(s); reported outcome(s); and main finding(s). Then we found all full-texts of the articles selected and the exclusion criteria were also applied to the full-texts.

We categorized the results from studies on sources of MEs using “framework of factors influencing clinical practice and contributing to adverse events” developed by Vincent C. in 2003 (11); we added an extra category entitled “medication” to what was suggested by Vincent.

We considered the results reported by more than 30% of studies in every category as the most frequent topic. We report the findings in different unit of observations based on their most frequent percentages.

## Results

**Figure 1:**
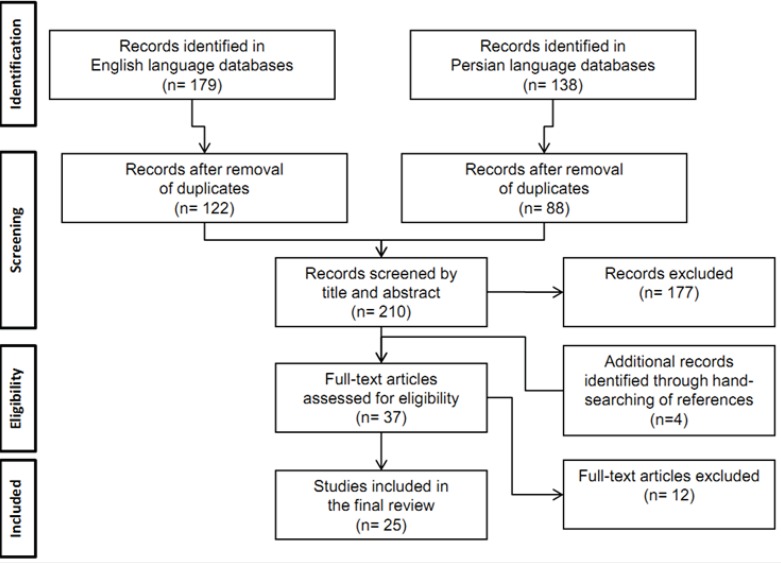
Search process and number of eligible studies

Initially, 122 and 88 studies were identified in English and Persian biomedical databases respectively, after removing the duplicates. Of the 210 studies, 177 were of no relevance to the current review according to their titles and abstracts. Twelve studies did not meet the inclusion criteria according to their full-text. After hand-searching of the reference lists of all primary studies, we added another 4 studies; this left us with 25 eligible studies for our final review. Figure 1 summarizes the complete process of selection.

Out of 25 studies, 12 of them reported sources of MEs, 11 assessed most frequent drugs involved in MEs, 5 focused on underreporting of and 5 on preventing measures of MEs.

The studies were published from 2007 onwards and all of them were conducted in hospital settings. Nursing staff and nursing students were the most frequent populations under observation. Except for 2 interventional studies, all other studies were cross-sectional.

*Number of relevant studies*

The 12 studies on sources of MEs investigated individuals (11), work environment (5), organization and management (4), team (4), tasks (2), and medications (2) as the possible causes of ME. Anti-infectives for systemic use (9) and cardiovascular system drugs (6) were the main drugs assessed in Iranian studies. Personal fears (5), reporting process (5) and administrative barriers (4), were the main areas of interest in studies focused on underreporting of MEs. Detailed characteristics of studies are summarized in Table 1.

**Table 1:** Characteristics of studies on ME included in our final review with their most frequent topics covered

**Categories of MEs**	**# of Studies**	**Publication Years**	Units of Observation(# of studies) *	**Study Design**	**Most Frequent Topics Covered ** (# of studies) **
Sources of MEs	12	2007-2012	Nursing staff (9) Nursing students (4) Nurse instructors (2) Hospital officials (1)	Cross-sectional	**Individuals (** 10 **)** Inadequate knowledge on medication (7) Miscalculation of dosages (6) Poor physical or mental health (4) **Work environment (**5**)** Heavy workload (4) Working over time (2) Nurses’ burnout (2) Little time spent with patient (2) **Organization and management (**4**)** Shortage of workforce (understaffing) (4) **Team (**4**)** Illegibility of orders or patient charts (4) **Tasks (**2**)** Lack of guidelines (2) **Medications (**2**)** Name similarity (2)
Under-reporting of MEs	5	2008-2012	Nursing staff (4) Nursing students (2) Personnel (1) Midwives (1)	Cross-sectional	**Fears (** 5 **)** Being reprimanded (4) Receiving negative attitudes from patients or family members (2) Experiencing adverse drug consequences for patients (2) Being recognized as incompetent (2) **Reporting process (**5**) ** Ignoring to report (5) Providing unclear MAE definition (4) Forgetting to report (3) **Administrative barriers (**4**) ** Focusing on individual rather than system factors to address MAEs (4) Lacking of positive feedbacks (3) Putting much emphasis on MAEs as quality indicator of nursing care (2)
Preventing measures of MEs	5	2008-2011	Nurses (1) Nurse instructors(1) Hospital officials (1) Physicians (1) IV administration by nurses (1)	Cross-sectional; Before – after clinical trial	****
Drugs involved in MEs	11	2007-2012	Nurses (1) Nursing students (4) IV administration by nurses (2) Administered doses by nurses (1) Infusion pump doses (1) Medical charts (1) Patients (1)	Cross-sectional; Non-randomized clinical trial	Anti-infectives for systemic use (9)*** Cardiovascular system (6)***

* A few studies included more than one units of observation. So, the total units of observations may surpass the total number of studies.

** Topics which had been covered in over 30% of studies were mentioned as most frequent.

*** Based on Anatomical Therapeutic Chemical (ATC) Classification System.

**** Inconsistency of topics and the diversity of reporting limited us to provide 3 most frequent topics in this category.

MAEs: Medication Administration Errors, IV: Intravenous.

*Sources of MEs*

Only one out of 12 studies (8.3%) was in English. All study designs were cross-sectional. Most, not all studies, in this category reported the frequency of each source of ME as percentages; but few of them mentioned the importance of each source using a Likert scale with scoring from 1-5 in which score 5 corresponded to the highest degree of importance of the corresponding source of MEs.

Individual factors were regarded as accountable in 27.7% to 79.9% of MEs by nurses. The corresponding range was from 15.3% to 29.7% in nursing students.

All four studies which were performed on nursing students reported individual factors as the main and only source of MEs occurrence. Miscalculations of doses (100% of studies) with estimated prevalence between 13.5% and 20.0%, and inadequate knowledge of medications (75% of studies) with estimated prevalence between 11.4% and 18.9% were reported as the most frequent contributing factors.

In studies on nurses’ views shortage of workforce was accounted as one of the most reported sources (4 out of 5 studies; 80%) with regard to “organization and management”. It had one of the highest prevalence in all contributing factors (12.8% to 100.0%). Heavy workload had the estimated prevalence between 10.6% and 70.0% in different contexts and was reported in 80% of studies. Nurses also reported physical and mental health as the most frequent source of MEs at individual level which ranged from 48.7% to 79.9% in prevalence. Illegible handwritings (15.0% to 70.0%) and inadequate knowledge of medications (27.6% to 55.8%) was reported by nurses as the next most frequent individual factors.

In general, the most commonly-reported contributory factor to ME was individual factors (10 of 12 studies) in which the inadequate knowledge of medication (7 of 12 studies) was the most frequent reported one. The three most-commonly reported individuals contributing factors to MEs in all studies were personal problems (48.7% to 79.9% in different studies), inadequate knowledge of medication (11.4% to 55.8%), and dose miscalculations (13.5% to 20.0%). 

We could not categorize two studies using Vincent’s framework; one of them assessed the association between nurses’ satisfaction from working conditions and frequency of their MEs (3) and the second, assessed sources of MEs from hospital officials’ viewpoint (6). The most frequent findings from studies on sources of ME are summarized in Table 2.

**Table 2:** Detailed characteristics of studies on sources of ME with their most frequent findings

**Findings**	**Sources of Medication Errors** **(In descending order)**	**Study Design and/ or Measurement Tool**	**Sample Size**	**Unit of Observation**	**Author(s) / Year**	
12.76% 4.2% 10.63% 14.89% 27.65% 19.14% 48.93% 23.4% 19.14%	**Organization and Management** Shortage of workforce Lack of training **Work environment** Heavy workload leading to fatigue **Team ** Patient’s charts illegibility** Individuals Inadequate knowledge of medication Miscalculation of doses **Medications ** Using abbreviations for drug names Name similarity Different drug doses	Cross-sectional; Self-report survey (Questionnaire)	64	Nurses	^(^^12^^)^Cheraghi MA, *et al*. 2012	1
Mean score SD 4.380.96 4.160.98 4.131.01 3.821.17 3.611.18 3.881.08 3.441.66 3.661.16 3.461.8	**Organization and management** Shortage of workforce **Work environment** Nurses burnout Heavy workload Little time spent with patient **Team ** Inadequate supervision on ward Physician’s handwriting illegibility** Patients’ charts illegibility **Individuals** Mental problems* Tasks Lack of guidelines	Cross-sectional; Self-report survey (Questionnaire)	200	Nurses	^(^^13^^)^ Hosseinzadeh M, *et al*. 2012	2
Mean score SD 4.150.57 4.410.58 4.170.49	**Team ** Orders illegibility **Individuals** Inadequate knowledge of medication Miscalculation of doses	Cross-sectional; Self-report survey (Questionnaire)	22	Nurse instructors	^(^^14^^)^Baghcheghi N, *et al*. 2010	3
29.72% 24.32% 13.51%	**Individuals** Documenting wrong medication in patients’ charts Failing to check drug dosage in patients’ charts Miscalculation of doses	Cross-sectional; Self-report survey (Questionnaire)	78	Nursing students	^(^^15^^)^ Mohammadnejad E, *et al*. 2010	4
62% 70% 58% 50% 59% 37% 35%	**Organization and management** Shortage of workforce **Work environment** Heavy workload Working overtimes Inadequate equipments **Individuals** Fatigue and exhaustion* Medications Similar packages Name similarity	Cross-sectional; Self-report survey (Questionnaire)	100	Nurses	^(^^16^^)^ Nikpeyma N, *et al*. 2009	5
100% 83.7% 83.7% 79.9% 69.8% 79.9% 64.0% 55.8%	**Organization and management** Shortage of workforce **Work environment** Frequent work shifts Working overtime, night-time and consecutive shifts Chaotic ward **Team ** Illegible handwriting **Individuals** Personal problems* Lack of experience Inadequate knowledge of medication	Cross-sectional; Self-report survey (Questionnaire)	86	Nurses	^(^^17^^)^Ghasemi F, *et al*. 2009	7
18.95% 18.79% 16.99%	**Individuals** Inadequate knowledge of medication Miscalculation of doses Lack of infusion monitoring	Cross-sectional; Direct observation	52	Nursing student	^(^^18^^)^ Baghcheghi N, *et al*. 2008	6
15.25% 13.55% 13.55%	**Individuals** Inadequate knowledge of medication Failing to check drug dosage in patients’ charts Miscalculation of doses	Cross-sectional; Self-report survey (Questionnaire)	76	Nursing student	^ (^^19^^)^Koohestani HR, *et al*. 2008	8
20.00% 14.28% 11.42%	**Individuals** Miscalculation of doses Failing to check drug dosage in patients’ charts Inadequate knowledge of medication	Cross-sectional; Self-report survey (Questionnaire)	60	Nursing student	^(^^20^^)^ Koohestani HR, *et al*. 2008	9
66.7% 42.1% 38.9% 48.7% 40% 30% 16.7%	**Work Environment** Nursing burnout Little time spent with patient Heavy workload **Individuals** Mental problems* Inadequate knowledge of medication Disappointment and lack of interest **Tasks** Lack of guidelines	Cross-sectional; Self-report survey (Questionnaire)	40	Nurses	^(^^21^^)^ Souzani A, *et al*. 2007	10
Studies which categorized sources of medication error un-comparable to classification by Vincent (11).						
Mean **36.1541.15** **22.2831.72** **9.3214.87** **5.23.1**	**Work conditions** **Very unsatisfactory ** Administering drug without physician’s order Administering drug before and after appropriate time Administering several oral drugs simultaneously **Unsatisfactory ** Administering several oral drug simultaneously Administering drug before and after appointed time Inappropriate time (before/ after meal) for administering drug **Satisfactory ** Administering several oral drug simultaneously Administering painkiller without physician’s order Administering drug before and after appointed time **Very satisfactory ** Administering several oral drug simultaneously Administering painkiller without physician’s order Administering drug before and after appointed time	Cross-sectional; Self-report survey (Questionnaire)	286	Nurses	^(^^10^^)^Joolaee S, *et al*. 2011	11
**7.29%** ^a^ 0.71^b^ 0.65^b^ 0.63^b^	**Drug information** No list of automatic stop orders medications in every ward No pharmacology textbooks in wards No registration of drug history on admission	Cross-sectional; Self-report survey (Questionnaire)	396	Hospital officials (12 person team)	^(^^22^^)^ Nasiripour AA, *et al*. 2011	12

^a ^Percentage of variance from factor analysis, ^b ^factor loadings

* Have been categorized as physical and mental health.

** Have been categorized as illegible orders or handwriting.

*Underreporting of MEs*

Personal fears (Likert score range: 3.5 to 4.1), administrative barriers (3.6 to 3.8) and the reporting process (1.6 to 3.1) were the barriers for reporting MEs.

Ignoring to report as the most frequent barrier in MEs reporting (100% 0f studies) had the importance score between 1.4 and 3.5. Fear of “decreasing evaluation scores and introducing educational problems” had the highest importance (4.4 to 4.6) which was reported by nursing students. Nurses also declared focus on individual rather than system factors to MEs as the most important barrier (score 4.0).

Just one study reported the frequency of barriers; 23.3% for personal fears, and 14.5% for ignoring the report in the reporting process (23).

The most frequent findings from studies on reasons for underreporting of MEs are summarized in Table 3.

**Table 3:** Detailed characteristics of studies on underreporting of ME with their most frequent findings.

**Findings**	**Reporting of Medication Errors ** **(descending order)**	**Study Design and/ or Measurement Tool**	**Sample Size**	**Unit of Observation**	**Author(s) / Year**	
Mean score **3.5** 3.9 3.9 3.5 **3.8** 4.0 3.9 3.8 **3.1** 3.3 3.0 2.9	**Fears** Being subject to lawsuit Experiencing adverse drug consequences for patients Being reprimanded by instructors* Administrative barriers Focus on individual rather than system factors to MAEs Disproportional responses to severity of errors Disproportional responses to importance of errors **Reporting process ** Providing unclear MAE definition Forgetting to report Ignoring to report	Cross-sectional; Self-report survey (Questionnaire)	200	Nurses	Hosseinzadeh M, *et al*. 2012 ^(^^13^^)^	1
Mean score - 3.8 3.3 3.2 - 3.9 3.7 3.7 - 3.2 2.9 2.6	**Fears** Being subject to lawsuit Experiencing adverse drug consequences for patients Awareness of other departments and centers on MAE by the nurse **Administrative barriers** Focusing on individual rather than system factors for MAEs Providing no positive feedbacks Instructors’ negative beliefs **Reporting process ** Forgetting to report Providing unclear MAE definition Ignoring to report	Cross-sectional; Self-report survey (Questionnaire)	140	Personnel	Tol A, *et al*. 2010^(^^24^^)^	2
23.3% - 14.5% 6.5%	**Fear of** Being reprimanded by nurse instructors **Reporting process ** Ignoring to report Quickly realizing errors	Cross-sectional; Self-report survey (Questionnaire)	332 68	Nurses; Midwives	Zahmatkeshan N, *et al*. 2010(23)	3
Mean score** 3.5 4.4 4.2 4.1 **3.6** 4.3 3.8 3.9 **2.5** 3.5 2.5 2.5	**Fears** Decreasing evaluation scores and introducing educational problems Being reprimanded by instructors Being recognized as incompetent **Administrative barriers** Providing no positive feedback Putting much emphasis on MAEs as quality indicator of nursing care Focusing on individual rather than system factors for MAEs **Reporting process ** Ignoring to report Spending too much time for contacting instructor Providing unclear MAE definition	Cross-sectional; Self-report survey (Questionnaire)	240	Nursing students	^4^ Koohestani HR, *et al*. 2009(25)	4
Mean score **4.1** 4.6 4.5 4.5 **3.6** 4.3 4.1 3.3 **1.6** 2.1 1.4 1.4	**Fears** Decreasing evaluation scores and introducing educational problems Being reprimanded* Experiencing adverse drug consequences for patients **Administrative barriers** Providing no positive feedback Putting much emphasis on MAEs as quality indicator of nursing care Focusing on individual rather than system factors for MAEs **Reporting process** Forgetting to report Ignoring to report Providing unclear MAE definition	Cross-sectional; Self-report survey (Questionnaire)	76	Nursing students	(26)Koohestani HR, *et al*. 2009	5

* All categorized as “being reprimanded”. ** The scoring scale used by Koohestani *et al.* ranged between 1 to 6 (25); we adjusted it to scales by other authors which ranged between 1 to 5.

*Preventing MEs*

We could not categorize the most frequent topics from studies on preventing MEs because of the diversity in reporting and the resultant inconsistencies in the findings. Details of findings from studies on preventing MEs are summarized in Table 4.

**Table 4:** Detailed characteristics of studies on preventing ME with their most frequent findings

**Findings **	**Preventing Medication Errors**	**Study Design and/ or Measurement Tool**	**Sample Size **	**Unit of Observation**	**Author(s) / Year**	
91.2% 60.5% 50.1% 75.6% 6.6% 27.6%	**Before educational intervention** Patient training Assessment before prescription Length of injection **After educational intervention** Patient training Assessment before prescription Length of injection	Non randomized clinical trial	603	IV administration by nurses	Sharifi N, *et al*. 2012 (27)	1
**7.28** **a** 0.717b 0.656b 0.6b	**Launching an electronic prescription system, proper medication labeling and packaging from pharmacy** Providing access to list of automatic stop orders medications in wards Providing access to pharmacology textbooks in wards Registration of drug history on admission by physicians	Cross-sectional; Self-report survey (Questionnaire)	396	Hospital officials (12 person team)	Nasiripour AA, *et al*. 2011(28)	2
40% 40% 35%	Increasing duration of theoretical education for pharmacology course Adapting educational objectives to practical requirements of students in pharmacology Promoting case methods for drug administration instead of functional methods	Cross-sectional; Self-report survey (Questionnaire)	22	Nurse instructors	Baghcheghi N, *et al*. 2010(14)	3
52% 33% 1% 1% 41% 22% 25% 20%	**Prescription errors ** Before intervention After CPOE + CDSS **Transcription errors ** Before intervention After CPOE + CDSS **Medication errors ** **Dose errors ** Before intervention After CPOE + CDSS **Frequency errors ** Before intervention After CPOE + CDSS	Before-after interventional study	248 5657	Patients Physicians Order	Kazemi A, *et al*. 2009(29)	4
98.8% 96.5% 69.8%	Increasing number of staff proportional to patient load Training personnel Giving information on new drugs	Cross-sectional; Self-report survey (Questionnaire)	86	Nurses	Ghasemi F, *et al*. 2009 (17)	5

CPOE: Computerized Physician Orders Entry; CDSS: Clinical Decision Support System

^a ^Percentage of variance in preventing nursing practice from factor analysis, ^b ^factor loadings

*Most common wards and most frequent drugs*

The most common wards under assessment for ME were the intensive care units and internal medicine wards followed by critical care units and surgery wards.

Three studies did not report any frequencies of MEs with each drug or drug class. The reporting approach of the remaining 8 studies was very diverse; most of them mentioned specific drug names and frequency of corresponding MEs and the others just stated the overall drug classes.

Drugs with the highest prevalence involved in MEs ever were intravenous fluids in a pediatric ward (76.2%) and albumin in an internal medicine ward (62.0%). But generally, antibiotics were the most common drugs involved in MEs in different studies; the range of ME frequencies differed according to the wards and study populations of interest. Estimation of MEs in antibiotics administration by nurses varied widely from 11.0% for amikacin in an intensive care unit to 56.4% for ceftazidime in an internal medicine ward; the corresponding figure for nursing students was 4.6% for ceftazidime in an internal medicine ward. Cardiovascular drugs (including heparin) usually followed antibiotics; overall, the range of MEs in cardiovascular drugs by nurses varied from 15.6% to 21.0%.

Table 5 summarizes detailed characteristics and findings of studies on most frequent drugs involved in MEs.

**Table 5:** Detailed characteristics of studies on most frequent drugs with ME and their findings

**Findings**	**Drugs**	**Type of Ward**	**Unit of Observation**	**Author(s) / Year**	
NA	Ceftriaxone Cefazolin Vancomycin	Endocrinology & nephrology Gastrointestinal & respiratory Neurology Infectious disease	Nursing students	Ebrahimi Rigi Tanha Z, *et al*. 2012(30)	1
23.5% 15.6% 13.7%	Antimicrobials Cardiovascular Gastrointestinal	Intensive Care Units (ICU)	IV administration by nurses	Vazin A, *et al*. 2012(31)	2
62.0% 56.4% 49.5%	Albumin Ceftazidime Metronidazole	Internal	IV administration by nurses	Sharifi N, *et al*. 2011(27)	3
38.4% 38.4%	Immunosuppressive anti-infective	Nephrology	Patients	Vessal G. 2010(32)	4
76.2% 11.3% 7.9%	Intravenous fluids Antibiotics Anti- inflammatories	Pediatric	Pediatrics’ medical charts	Mohsenzade A, *et al*. 2010(33)	5
NA	Aspirin Heparin Cefazolin	Emergency Cardiovascular Surgery Internal	Nursing students	Mohammadnejad E, *et al*. 2010(15)	6
27% 21%	Antibiotics Cardiovascular	ICU CCU Pediatric Internal Surgery Emergency	Nurses	Nikpeyma N, *et al*. 2008(16)	7
11.0% 8.2% 7.4% 7.4% 7.4% 7.4%	Amikacin Vancomycin Diazepam Metoclopramide Metronidazole Ranitidine	ICU	IV administration by nurses	Fahimi F, *et al*. 2008(34)	8
6.5% 4.6% 3.9% 3.9%	Heparin Ceftazidime Dopamine Phenytoin	Internal Surgery CCU Neurosurgery	Nursing student	Baghcheghi N, *et al*. 2008(18)	9
NA	Heparin	CCU	Nursing student	Koohestani HR, *et al*. 2008(20)	10
30% 30% 25%	Three NitroGlycerin Midazolam Dopamine	ICU	Infusion pump doses	Fahimi F, *et al*. 2007(35)	11

## Discussion

This review intended to detect and evaluate the studies on source of MEs, reasons for MEs under-reporting, preventive measures of MEs and the most common drugs related to MEs in Iran. It demonstrates the existing gaps and evidence insufficiency in the current published literature which were investigated and the need for improvement in different aspects of study designs for future. 

*Sources of ME*

Individual factors were the most frequently claimed source of ME occurrence reported in Iranian studies. This could be because MEs which are attributed to human failure are somewhat easier to recognize (3). But, in reality, they contribute to small percentage of MEs because system failures are the main cause in the vast majority of MEs (3, 4). Nursing students reported individuals as the main and only source of MEs occurrence with inadequate knowledge of medications, and miscalculations of doses as the most frequent factors. But based on nurses’ views, shortage of workforce, heavy workload, and physical and mental health problems were the main and most reported reasons. Illegible handwritings and inadequate knowledge of medications were recognized as the next most frequent sources of MEs. These differences between nursing students and nurses could be due to their personal skills and also institutional settings and responsibilities. 

In different studies, dose miscalculation (8, 36-39) and inadequate knowledge of medication (1-3, 5, 8, 36, 37, 39, 40) were considered responsible for the most incidents of ME. These have been reported frequently in the literature as one of the most common contributing factors (2). 

Nevertheless, it has been reported that nurses are at particular risk for making errors in calculating dosages because their mathematical skills are not well developed (8, 39, 41). Moreover, nurses routinely perform medication administrations; but, studies have revealed that they do not always have sufficient knowledge about the medication itself (1). Based on the systematic review by Alsulami *et al.*, poor knowledge of prescribed or administered medications was the most common reported contributory factor for MEs in Middle Eastern countries (5). 

As we stated in our results, shortage of workforce (8, 13, 39, 40, 42), heavy workload (2, 3, 39, 40, 42), poor physical or mental health (1, 40), are also common factors contributing to MEs in different studies and literature reviews. Shortage of workforce or increased workload can often lead to shifting highly-skilled staff from their standard actions (1). Staffing shortage also may cause an increase in administrative activities of nurses, which augments the chance of MEs (8, 39). 

Illegibility of physician orders (3, 8, 39, 40) is a frequent causative factor to MEs and sometimes is accounted as the main factor (36). Nurses frequently administer medications in an unsafe manner due to poor standard of written prescriptions (39). Physicians have the least readable handwritings (8, 39) which place nurses at risk of making errors in administering medications (39). 

These factors have not been assessed as frequent sources of ME in our included studies: inexperience (3, 5, 39, 40, 43), distraction (3, 8, 39, 40, 43) and interruption (1, 8, 36, 39) of nurses, and also medication characteristics such as similar names (3, 40) and large number of new drugs (3).

*Reasons for MEs underreporting*

Personal fears were the highest important reasons as the barrier for reporting in all of our studies. In the review by Aronson *et al.*, personal fears have been stated as major perceived barriers (37). 

Ignoring to report was the most frequent reason for underreporting (100% of the studies) in our review. It has been mentioned in other studies too with somehow similar phrases such as “error is not considered serious enough to report”, or “perception of non importance” (8, 43). 

Several authors have stated that fear of being reprimanded and punishment is the most frequent barrier (2-4, 6, 8, 9, 38, 43); in our study, this became as the second most frequent barrier. There are many studies which emphasize on non-punitive (2, 6), blame free (3, 8, 38) and supportive (8) work environment for ME reporting; otherwise, health care’s providers will not report coverable errors (2). This emphasis has lead to the need for an anonymous reporting system which is usually lacking in developing countries including Iran. 

*MEs prevention strategies*

Many preventive measures have been suggested in different overseas and Iranian studies like providing access to pharmacological text books (36, 38), defining protocols (44), formulary interchanges (42), launching electronic prescription systems (2, 6, 36, 37), medication labeling and packaging (2, 9, 37, 45), patient education about their treatment (4), physician education about appropriate guidelines for prescribing(46), providing information about new drugs (3, 36, 39) and compensating staff shortages (4). Some other approaches have also been declared to prevent MEs outside Iran such as avoidance of unsafe abbreviation (3, 37), national drug chart to reduce MEs related to documentation(3), national prescription forms (6), education of nurses in mathematics or calculation(3, 36), establishing formal ME reporting system (36, 38), supportive and non-punitive environment (3, 4), double checking (3), standardizing drug names(37), and checking five “right”s; right medication, patient, dose, route and time (3). 

In the systematic review based on studies from Middle Eastern countries, two types of interventional study have been reported; intervention by clinical pharmacists and the use of computerized physician order entry systems with or without clinical decision support (5). Pharmacist participation in drug rounds is an effective intervention, which leads to significant reduction in MEs (2, 5, 38, 47).

CPOE and/or CDSS are among the possible promising technologies such as bar codes (45) and personal digital assistants (3) that are expected to have positive effects on ME reduction (2, 3, 5, 6, 36-38, 45, 48). But, only one interventional study in our review had assessed the impact of CPOE and/or CDSS establishment on reducing MEs. Although many advantages and disadvantages of CPOE/CDSS technologies have been reported by other authors (3, 6, 37, 38, 48) (2, 3, 45), there seems a need for further studies to assess the feasibility and possibility of implementation in Iranian context. 

There is a huge lack of educational and interventional studies for preventing MEs. Considering the inability in generalization of this context based on its variation in different culture and countries, there is a need for these types of studies to evaluate different interventions in the Iranian context.

*Most frequent drugs involved in MEs*

We found much diversity in how studies reported most frequent drugs involved in MEs (3, 5, 38, 40, 48). The highest rate of MEs for administering intravenous fluids in a pediatric ward (76.2%) was much higher than study by Lewis* et al*. who reported the prevalence as 9% (48).In general, antibiotics were the most reported drugs involved in MEs in our studies; a finding which is in accordance with other reviews (3, 5, 38, 40, 48). The reported error rate for antibiotics by other studies was between 32% and 56% (40, 48); our studies reported a range between 11% and 56.4%. Cardiovascular drugs frequently followed antibiotics; other studies (5, 38, 40, 48) reported the same with the estimated prevalence of 16% to 17% (40, 48).

It is better to give the priority for further research to those drugs that carry more risk and are associated with more severe and frequent MEs.

## Conclusion

Based on our results and discussion, we may suggest further study topics to bridge the gap in research on ME. These topics are:

1. Design, implementation, and evaluation of a systematic ME reporting system;
2. Assessing systems-related factors to ME alongside individual factors;
3. Assessing other stakeholders of MEs including health care professionals such as physicians, pharmacists, undergraduate students, etc.;
4. Evaluating the effectiveness of preventive measures for MEs in trials; and
5. Evaluating the effectiveness of interventional approaches which increase ME reporting by organizations and health care professionals.
